# Comprehensive Geno- and Phenotyping in a Complex Pedigree Including Four Different Inherited Retinal Dystrophies

**DOI:** 10.3390/genes11020137

**Published:** 2020-01-28

**Authors:** Johannes Birtel, Martin Gliem, Kristina Hess, Theresa H. Birtel, Frank G. Holz, Ulrich Zechner, Hanno J. Bolz, Philipp Herrmann

**Affiliations:** 1 Department of Ophthalmology, University of Bonn, 53113 Bonn, Germany; martin.gliem@roche.com (M.G.); Kristina.Hess@ukbonn.de (K.H.); s4thbirt@uni-bonn.de (T.H.B.); Frank.Holz@ukbonn.de (F.G.H.); philipp.herrmann@ukbonn.de (P.H.); 2 Center for Rare Diseases Bonn (ZSEB), University of Bonn, 53113 Bonn, Germany; 3 Roche Pharma Research and Early Development, F. Hoffmann-La Roche Ltd., 4070 Basel, Switzerland; 4 Senckenberg Centre for Human Genetics, 60314 Frankfurt, Germany; u.zechner@senckenberg-humangenetik.de (U.Z.); h.bolz@senckenberg-humangenetik.de (H.J.B.); 5 Institute of Human Genetics, University Medical Center Mainz, 55131 Mainz, Germany; 6 Institute of Human Genetics, University Hospital of Cologne, 50931 Cologne, Germany

**Keywords:** molecular testing, genotype, phenotype, dystrophy, retina, NGS, retinitis pigmentosa, Stargardt disease, Waardenburg syndrome, congenital stationary night blindness

## Abstract

Inherited retinal dystrophies (IRDs) are characterized by high clinical and genetic heterogeneity. A precise characterization is desirable for diagnosis and has impact on prognosis, patient counseling, and potential therapeutic options. Here, we demonstrate the effectiveness of the combination of in-depth retinal phenotyping and molecular genetic testing in complex pedigrees with different IRDs. Four affected Caucasians and two unaffected relatives were characterized including multimodal retinal imaging, functional testing, and targeted next-generation sequencing. A considerable intrafamilial phenotypic and genotypic heterogeneity was identified. While the parents of the index family presented with rod-cone dystrophy and *ABCA4*-related retinopathy, their two sons revealed characteristics in the spectrum of incomplete congenital stationary night blindness and ocular albinism, respectively. Molecular testing revealed previously described variants in *RHO*, *ABCA4*, and *MITF* as well as a novel variant in *CACNA1F*. Identified variants were verified by intrafamilial co-segregation, bioinformatic annotations, and in silico analysis. The coexistence of four independent IRDs caused by distinct mutations and inheritance modes in one pedigree is demonstrated. These findings highlight the complexity of IRDs and underscore the need for the combination of extensive molecular genetic testing and clinical characterization. In addition, a novel variant in the *CACNA1F* gene is reported associated with incomplete congenital stationary night blindness.

## 1. Introduction

Inherited retinal dystrophies (IRDs) are a major cause of severe visual impairment and blindness in the working-age population and contribute to a significant reduction of patients’ quality of life [1,2]. IRDs are characterized by a high clinical and genetic heterogeneity and a broad spectrum of symptoms, including progressive deterioration of visual acuity, reading difficulties, glare, color vision abnormalities, dark vision problems, and visual field defects.

With the development of novel therapeutic approaches—in particular gene therapy—a precise phenotypic characterization including multimodal retinal imaging as well as the detection of the disease-causing gene are becoming increasingly important [3]. Strengths of multimodal retinal imaging include the ability to detect metabolic and structural changes ahead of funduscopic visibility and to precisely monitor disease progression or potential therapeutic effects over time [3].

The development of high-throughput DNA sequencing methods has made it more feasible to also achieve diagnoses on a molecular level. Targeted Next-Generation Sequencing (NGS) in particular has been proven as an efficient tool and allows IRD differentiation according to their genetic causes in daily practice [4,5,6,7,8,9,10,11,12,13,14,15,16]. One strength of NGS is that a large variety of genes associated with IRDs can be studied simultaneously at relatively low cost.

Herein, we demonstrate the potential of combining comprehensive clinical characterizations and molecular genetic testing using targeted NGS in a complex pedigree. Four different phenotypes caused by mutations in independent genes were revealed in one family. Moreover, a novel variant in the *CACNA1F* gene is reported associated with incomplete congenital stationary night blindness (CSNB).

## 2. Materials and Methods

This retrospective, cross-sectional study was in adherence to the declaration of Helsinki. Institutional review board approval (Ethics Committee, Medical Faculty, University of Bonn) and patients’ informed consent were obtained. All subjects were identified in a referral center for rare retinal diseases at the Department of Ophthalmology, University of Bonn, Germany.

A general medical and ocular history was obtained from each patient and a standardized clinical examination was performed. Clinical assessment included standardized anterior segment and dilated fundus examination, best corrected visual acuity (BCVA) testing, and full-field electroretinography (ERG). Retinal imaging consisted of spectral domain optical coherence tomography (OCT), autofluorescence (AF) imaging with blue excitation light (both, Spectralis HRA+OCT, Heidelberg Engineering, Heidelberg, Germany), fundus photography (Visucam, Zeiss, Oberkochen, Germany) and wide-field pseudo-color- and AF fundus imaging (Optos PLC, Dunfermline, United Kingdom).

Genetic testing was performed as described previously. In brief, NGS analysis was performed by target enrichment with IDT xGen^®^ Inherited Diseases Panel v1.0 (IDT Integrated Technologies, Coralville, IA, USA), sequencing on an Illumina NextSeq 500 system (Illumina, San Diego, CA, USA) and data evaluation with the SeqNext module of the SeqPilot software (JSI Medical Systems, Ettenheim, Germany) [17].

## 3. Results

**Figure 1:**
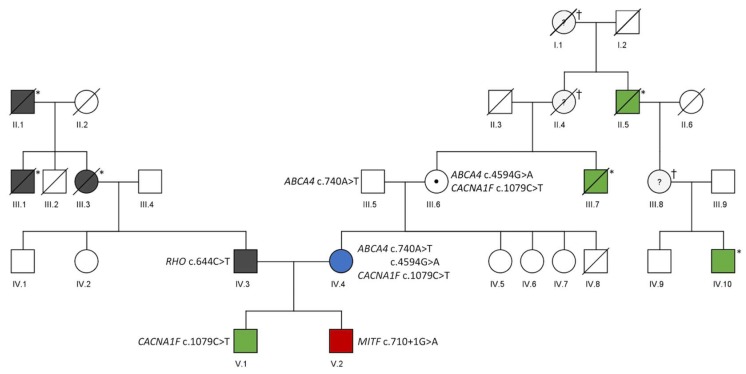
Genetic data of a pedigree including four different inherited retinal dystrophies. Patients with pathological findings are indicated by filled symbols. Black: *RHO*-related retinopathy. Green: *CACNA1F*-related retinopathy. Red: *MITF*-related retinopathy. Blue: *ABCA4*-related retinopathy. For individuals who underwent genetic testing, the identified variants are given next to the individual’s symbol. *: affected based on family history, †: assumed carrier of *CACNA1F*variant.

A summary of patient characteristics including identified mutations and a pedigree are provided in Table 1; Table 2 and Figure 1, respectively. The Caucasian family initially presented for an ophthalmological examination of their older son (V.1). Family history showed that other family members also revealed inherited retinal diseases that differed from each other and were not comprehensively characterized. Phenotypic similarities between the two sons (V.1, V.2) initially suggested the same genetic disease entity with different severity. However, molecular testing identified independent disease causes.

**Figure 2:**
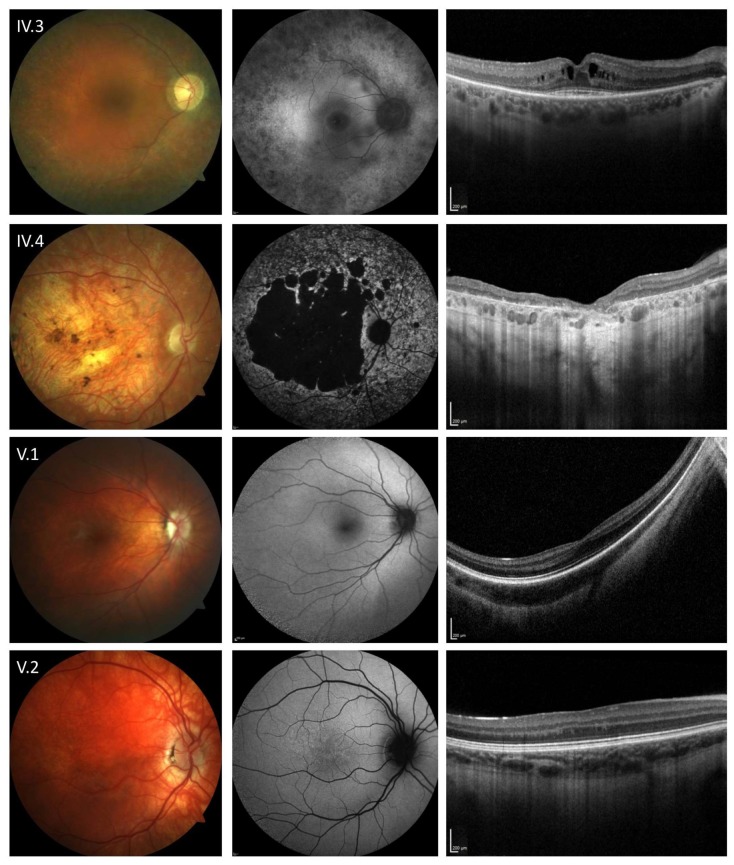
Retinal phenotype associated with mutations in the *RHO* (IV.3), *ABCA4* (IV.4), *CACNA1F* (V.1) and *MITF* (V.2) gene. Fundus color image (first column), fundus autofluorescence imaging with 488 nm excitation light (second column), and horizontal spectral-domain optical coherence tomography line scan (third column) are shown. Patient numbers refer to Figure 1. Only one eye is shown due to high symmetry between eyes.

The 48-year-old father (IV.3) reported dark adaptation problems since childhood and visual field defects starting in his second decade. At the age of 18, he was diagnosed with retinitis pigmentosa (RP) and a reduction of visual acuity was noted over the last years. Now, BCVA was 20/32 and 20/50 in the right and left eye, respectively. The deceased mother (III.3) of the patient as well as the maternal grandfather (II.1) were also affected by RP (Figure 1). On clinical examination (Figure 2) typical RP characteristics were revealed including bilateral posterior subcapsular cataract, arteriolar attenuation, retinal pigmentary changes, vitreous cells, a paracentral ring of increased AF, reduced AF in the (mid-) peripheral retina, and a cystoid macular edema on OCT imaging. Scotopic and photopic responses on ERG recordings were nearly extinguished. In line with these clinical findings, molecular testing identified a previously described heterozygous pathogenic missense variant (c.644C>T, p.Pro215Leu, Table 1; Table 2) in the *RHO* gene (NCBI Reference Sequence NM_000539.3) [18]. 

The 45-year-old mother (IV.4) reported reduced visual acuity and reading problems starting at the age of 15. One year later, she was diagnosed with macular dystrophy and over the last decades, she experienced a slow deterioration of visual acuity as well as pronounced glare and dark adaptation problems. At presentation, BCVA was hand movements in both eyes. Scotopic and photopic responses on ERG recordings were reduced. Ophthalmoscopy and retinal imaging revealed features of *ABCA4*-related retinopathy including pronounced bilateral chorioretinal atrophy, pigmentary alterations as well as abnormally in- and decreased AF surrounding the area of atrophy (Figure 2). Molecular testing identified two previously described causative heterozygous missense variants (c.740A>T, c.4594G>A, Table 1; Table 2) in the *ABCA4* gene (NCBI Reference Sequence NM_000350.3) in compound heterozygous state (confirmed by segregation analysis of her parents III.5 and III.6) [19,20,21].

The 13-year-old son (V.1) reported reduced visual acuity, reading problems, dark adaptation problems, photophobia since childhood, and no color vision problems. Horizontal nystagmus was present since the first weeks of life and visual acuity was described as stable since childhood. At presentation, BCVA was 20/200 and 20/80 in the right and left eye, respectively. Ophthalmoscopy revealed a nearly unremarkable fundus with only minor granular alterations and on OCT imaging foveal hypoplasia was noted (Figure 2). Electronegative scotopic responses with a reduced but measurable b-wave were identified on ERG. Clinical differential diagnosis included ocular albinism, achromatopsia, and CSNB and the latter was supported by the identification of a novel hemizygous missense variant (c.1079C>T, Table 1) in the *CACNA1F* gene (NCBI Reference Sequence NM_005183.4) resulting in an amino acid exchange at an evolutionarily highly conserved position (p.(Ser360Phe)) in the α_1F_ subunit of the Ca_v_1.4 calcium channel. In silico assessment using eight algorithms (DANN, FATHMM, FATHMM-MKL, LRT, MutationAssessor, MutationTaster, PROVEAN and SIFT [17]) predicted the variant to be pathogenic and affect the channel function in a deleterious way (Table 2). The variant has not been described in previous reports nor in clinical (HGMD, ClinVar, LOVD) or population (gnomAD Ex.) variant databases (Table 2). No further disease-relevant variants or CNVs were detected in currently known IRD-associated genes. Segregation analysis identified the mother (IV.4) as carrier for this variant. After the genetic test result was discussed with the family, the mother (IV.4) disclosed that her maternal uncle (III.7), granduncle (II.5) and a grandson (IV.10) of this granduncle also manifested symptoms in the spectrum of incomplete CSNB including nystagmus and non-progressive reduced visual acuity. However, they were deceased or not available for clinical examination or segregation analysis.

The 11-year-old son (V.2) reported reduced visual acuity, reading problems, and glare since childhood but no dark adaptation problems. At presentation, BCVA was 20/50 and 20/40 in the right and left eye, respectively. Ophthalmic examination and retinal imaging revealed microstrabism, iris heterochromia, iris translucence, fundus hypopigmentation with central granular alterations, and foveal hypoplasia (Figure 2). On ERG electronegative scotopic responses were identified. Furthermore, congenital sensorineural deafness, treated with cochlear implants, was reported. No further family members reported hearing problems. These clinical findings also pointed to the spectrum of ocular albinism, achromatopsia or CSNB. Molecular testing revealed a previously described, heterozygous pathogenic variant (c.710+1G>A, Table 1) in the *MITF* gene (NCBI Reference Sequence NM_000248.3) that has been associated with autosomal dominant Waardenburg syndrome [22]. This variant alters the consensus donor splice site sequence of intron 7 of the *MITF* gene. In silico assessment using six prediction algorithms (DANN, FATHMM-MKL, MutationTaster, Human Splicing Finder, Splice Site Prediction by Neural Network and varSEAK Online splice prediction tool [17]) predicted the variant to be a pathogenic null variant (Table 2). Another variant (c.710+1G>T) at the same base position has been also described as a pathogenic variant in patients with Waardenburg syndrome [23]. Segregation analysis indicated that the variant most probably occurred de novo in the patient. In the NGS analysis of currently known IRD-associated genes, no other disease-relevant variants or CNVs were detected. 

## 4. Discussion

Inherited retinal dystrophies are clinically and genetically heterogeneous diseases with currently more than 250 associated genes (RetNet, https://sph.uth.edu/retnet). In this report, we demonstrate that the combination of high-resolution retinal imaging, clinical, and functional examinations, as well as molecular testing is highly effective to characterize families with complex pedigrees including different IRDs and inheritance modes. This included the identification of a novel variant in the *CACNA1F* gene and previously described mutations in the *RHO*, *ABCA4*, and *MITF* genes.

While the diagnoses of the two unrelated parents (IV.3, IV.4), with autosomal dominant RP and autosomal recessive *ABCA4*-related retinopathy, respectively, were straight forward, the different molecular genetic findings of the two children (V.1, V.2) were rather unexpected. Both sons displayed characteristics seen in the phenotypic spectrum of ocular albinism, achromatopsia, CSNB even though distinct differences were identified. A conclusive disease classification was challenging at the time of the presentation and molecular genetic testing was required for precise disease classification. The phenotypes were in line with the molecular genetic findings and included independent retinal dystrophies with autosomal dominant de novo and X-linked variants, respectively. 

Mutations in the *RHO* gene, encoding for rhodopsin, the visual pigment initiating the phototransduction cascade in rod photoreceptors, are among the major causes for autosomal-dominant RP [12,24,25,26,27]. The known phenotypic variability, with some mutations leading to a diffuse rod-cone dysfunction while others cause a rather restricted phenotype primarily affecting the inferior hemisphere of the retina, has been associated with variable allelic expressivity [28,29]. 

*ABCA4*-related retinopathy, one of the most common monogenic causes for retinal degeneration, is known for a broad phenotypic spectrum, ranging from mild Stargardt disease to severe cone–rod dystrophy [11,30]. It is caused by biallelic mutations in the *ATP-binding cassette sub-family A member 4 (ABCA4*) gene [31]. Dysfunction or loss of function of the encoded protein product leads to excessive accumulation of visual cycle end-products such as lipofuscin in the retinal pigment epithelium (RPE), and eventually RPE and photoreceptor atrophy with associated vision loss [32,33,34,35].

Mutations in the X-linked *CACNA1F* gene, encoding a α1-subunit of a voltage-gated L-type calcium channel expressed in the photoreceptor synapses, in most cases lead to incomplete CSNB [36,37,38]. Each α1-subunit contains four repeat domains with six transmembrane helical domains [37]. The novel *CACNA1F* variant identified in this report results in an amino acid exchange (p.(Ser360Phe)) at an evolutionarily highly conserved position and is predicted to be pathogenic by all applied in silico algorithms. Taking these findings and the clinical diagnosis of incomplete CSNB into account, it is likely that this novel variant represents a disease-causing mutation.

Mutations in the *MITF* gene, encoding the Microphthalmia-associated transcription factor which plays an important role in the regulation of tyrosinase, a key enzyme for melanogenesis and melanocyte differentiation, are known to cause Waardenburg syndrome type 2A (WS2) and Tietz albinism-deafness syndrome (TS), two overlapping disorders [39,40,41,42,43,44]. While non-truncating mutations in the MITF basic domain lead to TS, encompassing more generalized effects including albinoid-like hypopigmentation of the skin, hair, iris and severe hearing loss, other mutations in the *MITF* gene cause WS2 characterized by patchy depigmentation and uni- or bilateral deafness [43,45]. The presented patient with patchy pigmentations, iris heterochromia and a predicted null variant was assigned to the spectrum of WS2.

## 5. Conclusions

In summary, we demonstrate the coexistence of four different IRDs caused by distinct mutations and inheritance modes in one family. We reinforce the complexity of IRDs and highlight the need for the combination of extensive molecular diagnostic and clinical characterization. A precise characterization may not only help to identify the correct diagnosis with a clear prognosis, but it also supports patient counseling and may have implications in view of future therapeutic options. In addition, we report a novel variant in the *CACNA1F* gene associated with incomplete CSNB.

## Figures and Tables

**Table 1:** Patient Characteristics.

ID	Age	Gender	Refraction [dpt] (sph/cyl)	OD OS	BCVA	ODOS	Gene	Zygosity	Exons/ Introns	Nucleotide	Protein	Reference
IV.3	48	m	−0.75/−2.25 −0.75/−1.00		20/32 20/50		*RHO*	het	Exon 3	c.644C>T	p.Pro215Leu	[18]
IV.4	45	f	+3.00/−2.25 +3.25/−2.50		HM HM		*ABCA4*	het	Exon 6	c.740A>T	p.(Asn247Ile)	[19]
								het	Exon 31	c.4594G>A	p.(Asp1532Asn)	[20,21]
							*CACNA1F*	het	Exon 8	c.1079C>T	p.(Ser360Phe)	novel
V.1	13	m	−1.75/−4.25 −2.75/−3.25		20/50 20/40		*CACNA1F*	hem	Exon 8	c.1079C>T	p.(Ser360Phe)	novel
V.2	11	m	+4.50/−0.50 +6.00/−1.25		20/200 20/80		*MITF*	het	Exon 7	c.710+1G>A	splice site	[22]

f: female, m: male, OD: right eye, OS: left eye, BCVA: best corrected visual acuity, HM: hand motion, het: heterozygous, hem: hemizygous.

**Table 2:** Minor allele frequencies and pathogenicity scores of the identified variants.

ID	Gene	Nucleotide Change	Protein Change	MAF (GnomADEx.)	DANN	FATHMM	FATHMM-MKL	LRT	Mutation Assessor	Mutation Taster	PROVEAN	SIFT	HSF, SSPNN, varSEAK Online	Reference
IV.3	*RHO*	c.644C>T	p.Pro215Leu	absent	0.9989	Tolerated	Damaging	Deleterious	High	Disease causing	Damaging	Damaging		[18]
IV.4	*ABCA4*	c.740A>T	p.(Asn247Ile)	absent	0.9905	Damaging	Damaging	Neutral	Medium	Disease causing	Damaging	Damaging	-	[19]
		c.4594G>A	p.(Asp1532Asn)	0.000107	0.9993	Damaging	Damaging	Deleterious	Medium	Disease causing	Damaging	Damaging	-	[20,21]
IV.4, V.1	*CACNA1F*	c.1079C>T	p.(Ser360Phe)	absent	0.9953	Damaging	Damaging	Deleterious	Medium	Disease causing	Damaging	Damaging	-	novel
V.2	*MITF*	c.710+1G>A	p.?	absent	0.9956	-	Damaging	-	-	Disease causing	-	-	LOF of donor splice site	[22]

MAF = minor allele frequency; LOF = loss of function.
